# Does fluid loading influence measurements of intestinal permeability?

**DOI:** 10.1186/cc3511

**Published:** 2005-03-21

**Authors:** Ilkka Parviainen, Jukka Takala, Stephan M Jakob

**Affiliations:** 1 Consultant, Department of Anesthesiology and Intensive Care, Kuopio University Hospital, Kuopio, Finland; 2 Professor, Department of Intensive Care Medicine, University Hospital Bern, Bern, Switzerland; 3 Consultant, Department of Intensive Care Medicine, University Hospital Bern, Bern, Switzerland

## Abstract

**Introduction:**

Urinary recovery of enterally administered probes is used as a clinical test of intestinal mucosal permeability. Recently, evidence has been provided that the recovery of some but not all sugar probes is dependent on the amount of diuresis and renal function. The aim of this study was to assess the effect of fluid loading on the urinary recovery of sugar probes in healthy volunteers.

**Methods:**

In a cross-over study, 10 healthy volunteers ingested 100 ml of a solution containing 0.2 g of 3-*O*-methyl-D-glucose (3-OMG), 0.5 g of D-xylose, 1.0 g of L-rhamnose, and 5.0 g of lactulose on two different days. The volunteers were randomized to receive either 2 litres of Ringer acetate or no fluid during the following 3 hours. The sugar concentrations were measured in 5-hour urine samples period.

**Results:**

Fluid loading increased urine production and urinary recovery of xylose. Fluid loading did not influence the urinary recovery of 3-OMG, L-rhamnose, or lactulose. Neither the lactulose/rhamnose ratio nor the 3-OMG/rhamnose ratio changed.

**Conclusion:**

Fluid loading increases mediated carbohydrate transport but not the lactulose/rhamnose ratio, after oral sugar administration in healthy volunteers. It remains to be determined whether sugar probes are handled differently in response to fluids in patients with organ dysfunctions.

## Introduction

Mucosal permeability is one of the few functions of the gastro-intestinal tract that can be quantified in the clinical setting. Increased intestinal permeability in critically ill patients has been associated with endotoxemia and remote organ failure [1]. Mucosal intestinal permeability can be assessed noninvasively by measuring urinary excretion of orally administered test substances. Typically, this involves estimation of the urinary recovery of single or multiple probes administered orally. Quantifying the absorption of two sugars of different sizes offers advantages compared with the use of single probes: urinary recovery of these probes expressed as a ratio is particularly sensitive because this can reflect the contrasting effects of decreased absorption of monosaccharides, such as rhamnose or mannitol, owing to the reduced surface area and increased permeability for larger disaccharides, such as lactulose or cellobiose, owing to the opening of intracellular pathways. A further advantage of this ratio is the elimination of errors due to non-mucosal factors, because variables such as rate of gastric emptying, intestinal transit, impairment of renal function, and completeness of urinary collection should affect both sugars similarly [2].

In experimental animals, fluid loading increased the urinary recovery of intravenously administered lactulose but not rhamnose, and thereby increased the lactulose/rhamnose (L/R) ratio [3], suggesting changes in tissue distribution or altered renal handling of the sugar probes. In critically ill patients with organ dysfunction, recoveries of cellobiose, sucrose, and mannitol were positively related to urinary volume [4]. If fluid loading and the related increase in diuresis effectively alters the recovery of some or all sugar probes in humans, this method might no longer be considered a valid test for the assessment of intestinal permeability. The aim of this study was to test whether intravenous fluid loading increases the urinary recovery of lactulose or other orally administered sugars in healthy volunteers.

## Methods

**Figure 1:**
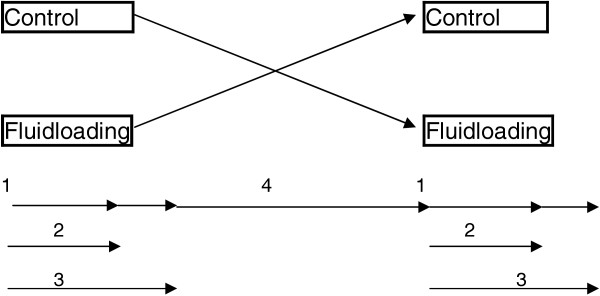
Experimental procedure for oral administration of sugar probes and fluid loading. 1, oral sugar administration; 2, 3 hours of fluid loading; 3, 5 hours of urine collection; 4, washout period from 4 to 6 days.

Ten healthy volunteers (four female, six male) with a median age of 32 years (range 23 to 42 years) were studied on the morning after an overnight fast. The subjects were not allowed to consume alcoholic beverages or to use drugs the day before the testing days. The study was approved by the Ethics Committee of Kuopio University Hospital. The research was performed in accordance with the Declaration of Helsinki. All volunteers gave written informed consent to participate in the study. In a cross-over design, the volunteers ingested 100 ml of a solution containing 0.2 g of 3-*O*-methyl-D-glucose (3-OMG), 0.5 g of D-xylose, 1.0 g of L-rhamnose, and 5.0 g of lactulose on two different days (Fig. 1). The volunteers were randomized to receive either 2 litres of Ringer acetate or no fluid during the following 3 hours. Before the ingestion of the sugar probes, the volunteers voided their urinary bladders. The sugar concentrations were measured at the end of the 5-hour urine sample period. The experiment was repeated after 4 to 6 days. Urinary recovery of the sugar probes assesses active (OMG) and passive (D-xylose) carrier-mediated, and nonmediated (L-rhamnose) transcellular absorption [2,5]. Lactulose permeates enterocytes through intercellular tight junctions [6]. The L/R ratio was calculated as an indicator of gut permeability. Urine samples were stored at -20°C until analysed by high-pressure liquid chromatography with pulsed amperometric detection [7]. The urinary recovery of each sugar probe was expressed as a percentage of the orally administered dose.

The data were analysed with Wilcoxon signed-rank test. Data are presented as medians and ranges.

## Results

**Figure 2:**
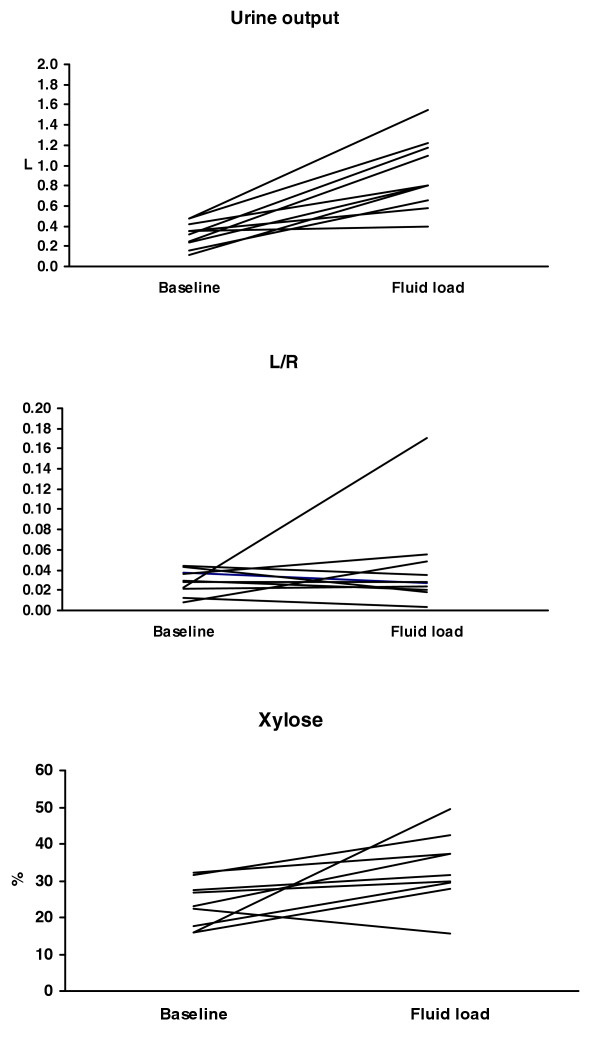
Urine output, L/R ratio and urinary recovery of xylose in individual volunteers.

Fluid loading increased urine production during the 5-hour collection period (Table 1, Fig. 2). Fluid loading did not influence the urinary recovery of 3-OMG, L-rhamnose, or lactulose. Neither the L/R ratio (Fig. 2) nor the 3-OMG/rhamnose ratio changed during fluid loading. The urinary recovery of xylose increased during fluid loading (Fig. 2).

## Discussion

Altered intestinal permeability has frequently been reported in patients requiring intensive care after major surgery and in sepsis. The mechanisms of these alterations are not entirely defined. In addition to permeability of intestinal epithelium, both pre-mucosal and postmucosal factors can affect the urinary excretion of enterally administered sugar probes [3,8]. Fluid loading has been reported to modify the excretion of sugar probes in rats [3]. Fluid loading is a common intervention in patients in intensive care. Altered excretion of sugar probes by fluid loading would invalidate the measurement of intestinal permeability in patients requiring rapid intravenous infusions of fluids.

We studied the effect of a postmucosal factor, fluid loading, on the urinary excretion of enterally administered sugar probes in healthy volunteers. In our study, only the urinary recovery of xylose increased after fluid loading. These results suggest that fluid loading does not affect measures of intestinal permeability, assessed by the L/R ratio, but increases mediated carbohydrate transport in healthy volunteers, measured by xylose recovery.

In contrast with our findings, fluid loading has been shown to increase the L/R ratio, caused by increased urinary lactulose excretion, in both control and endotoxemic rats [3]. Increased urinary lactulose recovery was observed both after intragastric and intravenous administration of the sugar probes. Together with the increased urine flow this suggests changes in the renal handling of lactulose during fluid loading [3]. In our study, despite increased urine flow during fluid administration, lactulose recovery did not increase. Proposed renal mechanisms for increased urinary lactulose recovery are a decrease in net proximal tubular Na^+ ^reabsorption and decreased pericapillary osmotic pressure with saline infusion, which might enhance the net movement of lactulose from the peritubular capillary into the lumen [9,10]. Systemic and renal handling of lactulose might be different in rats and humans. Nevertheless, we found a similar recovery rate of lactulose, rhamnose, and 3-OMG within a comparable urine collection period (5 hours versus 6 hours). In our study, the L/R ratio is also comparable with the ratios from other studies in healthy subjects [6,11,12].

In rats, Hallemeesch and colleagues [3] used double the amount of the daily fluid intake for fluid loading within 8 hours. This compares well with the 2 litres we infused during 3 hours in our volunteers. It is unlikely that the method of fluid administration (twice subcutaneously in the rats versus continually intravenously in human volunteers) influenced the results. Saline was used in rats, whereas we used Ringer acetate for fluid loading. The amounts of sodium and chloride are higher in saline, which might have influenced the different results from the two studies.

It has been shown in volunteers that renal clearance of lactulose but not of rhamnose is dependent on the intravenously administered quantity of the respective sugar [12]: urinary lactulose recovery was significantly lower after intravenous administration of a high dose in comparison with the regular dose. Although lactulose cannot enter the cells and therefore has an extracellular distribution, the monosaccharides might be present both intracellularly and extracellularly. Because the infusion of saline and Ringer solution increases the extracellular space, it is conceivable that crystalloid infusion might affect blood lactulose concentrations more than blood concentrations of the other sugars. Our results suggest that clinically relevant amounts of crystalloid infusion do not induce major alterations in concentrations of lactulose in the plasma.

Xylose and 3-OMG are used to measure the mediated transport of carbohydrates and to assess the intestinal absorptive capacity. In animals, D-xylose is transported across membranes by a Na-dependent transport [13]. However, the precise mechanism for D-xylose transport across human epithelial cells might differ from that described in animals [14]. Two possible routes are suggested: Na-dependent carrier-mediated entry and a paracellular shunt pathway [14]. In our study, the recovery of xylose increased during fluid loading. There was also a minor, but nonsignificant, increase in urinary recovery of 3-OMG. It is possible that fluid loading increases absorptive capacity by increasing cardiac output and intestinal mucosal perfusion.

In critically ill patients, sugar absorption tests as a measure of gastrointestinal permeability have been challenged. In patients with multiple organ failure, urinary flow and creatinine clearance were the most important determinants of urinary sugar recovery [4]. It was observed that renal dysfunction limits the excretion of cellobiose and sucrose but not that of mannitol. In addition, the recovery of disaccharides and mannitol decreased when urinary flow was lower. The authors concluded that a differential sugar absorption test is not a reliable measure of gastrointestinal permeability in patients with multiple organ failure. Because the relation between urinary recovery of the sugar probes and renal function and diuresis was analysed only post hoc, it cannot be excluded that renal dysfunction coexists with maintained gastrointestinal permeability in critically ill patients.

## Conclusion

Our results suggest that fluid loading despite increasing mediated carbohydrate transport does not affect the L/R ratio after oral sugar administration in healthy volunteers. However, further studies on intestinal and renal sugar handling in the assessment of gastrointestinal permeability are needed in critically ill patients.

## Key messages

• 	Fluid loading increases mediated intestinal carbohydrate transport in healthy volunteers.

• 	Fluid loading does not affect intestinal permeability as assessed by the lactulose/rhamnose ratio in healthy volunteers.

## Abbreviations

3-OMG = 3-O-methyl-D-glucose; L/R ratio = lactulose/rhamnose ratio.

## Competing interests

The author(s) declare that they have no competing interests.

## Authors' contributions

IP analysed data and wrote the final manuscript. JT designed the study and participated in the drafting of the manuscript. SJ recruited subjects into the study, managed them and participated in the drafting of the manuscript. All authors read and approved the final manuscript.

## Figures and Tables

**Table 1:** Urine volumes, urinary recoveries of sugar probes, L/R ratio and 3-OMG/R ratio.

Parameter	Without fluid load	With fluid load	Wilcoxon SR test
Urine volume, ml	300 (10–500)	800 (400–1600)	0.005
3-OMG, %	44 (29–63)	51 (40–77)	0.074
Rhamnose, %	7 (4–14)	11 (3–22)	0.169
Xylose, %	25 (16–42)	34 (16–50)	0.017
Lactulose, %	0.2 (0.1–0.3)	0.3 (0.0–0.6)	0.074
L/R ratio	0.03 (0.01–0.05)	0.03 (0.00–0.17)	0.878
3-OMG/R ratio	6.1 (3.3–9.6)	4.8 (3.0–14.5)	0.878

Values are median (range). 3-OMG, 3-*O*-methyl-D-glucose; 3-OMG/R, 3-*O*-methyl-D-glucose/rhamnose; L/R, lactulose/rhamnose; SR, signed-rank.
